# Catch-up growth following early-life stunting in a low-resource area in rural Tanzania: the MAL-ED Metabolic study

**DOI:** 10.1136/bmjopen-2025-100955

**Published:** 2025-08-21

**Authors:** Kiya Nemati, Yotham Z Michael, Bernadetha P Hhando, Samwel Jatosh, Eric R Houpt, Estomih Mduma, Mark D DeBoer

**Affiliations:** 1 Pediatrics, University of Virginia School of Medicine, Charlottesville, Virginia, USA; 2 Centre for Global Health, Haydom Lutheran Hospital, Mbulu, Tanzania, United Republic of; 3 Haydom Lutheran Hospital, Mbulu, Tanzania, United Republic of; 4 Medicine, University of Virginia School of Medicine, Charlottesville, Virginia, USA

**Keywords:** Paediatric endocrinology, NUTRITION & DIETETICS, Tropical medicine

## Abstract

**ABSTRACT:**

**Background:**

Poor linear growth over time can lead to stunting, a significant public health problem in low-resource settings. Catch-up growth, the process of accelerated growth following growth faltering, is important for mitigating the long-term impacts of early stunting. This study aimed to identify key predictors of growth over time, stunting and catch-up growth among children in rural Tanzania.

**Methods:**

We evaluated 182 children from the Etiology, Risk Factors, and Interactions of Enteric Infections and Malnutrition and the Consequences for Child Health and Development cohort, whose anthropometric measurements were collected at six points from birth to 11.5 years. We assessed outcomes of height-for-age z-score (HAZ), stunting and catch-up growth using mixed-model linear and logistic regression to assess associations of maternal education, household income, socioeconomic status, insulin-like growth factor 1 (IGF-1) and thyroid function tests. We defined stunting as HAZ ≤−2 and catch-up growth both as stunting resolved from age 2 years to 11.5 years and a HAZ increase of >0.5 from 2 years to 11.5 years.

**Results:**

Cohort participants exhibited a moderate amount of catch-up growth, with per cent stunting decreasing from 72.6% at 2 years to 39.0% at 11.5 years. Maternal education, household income, socioeconomic status and IGF-1 were positively associated with HAZ (eg, IGF-1 point estimate 0.141±0.067, p=0.036) and negatively associated with odds of stunting across time points, while thyroid-stimulating hormone was negatively associated with HAZ and positively associated with odds of stunting (all p<0.05). For the definitions of catch-up growth, only IGF-1 was associated with odds of catch-up growth by both definitions (both p<0.01). Because of the potential for IGF-1 to be associated with maturation (and potential early growth cessation), we also assessed regression models that included IGF-1, luteinizing hormone and follicle-stimulating hormone, revealing that IGF-1 remained associated with catch-up growth (p<0.05).

**Conclusions:**

These findings highlight the need for comprehensive interventions that address socioeconomic, hormonal and biological factors to promote catch-up growth and reduce stunting in resource-limited settings. The results offer valuable insights towards improving child health outcomes in similar contexts.

**Trial registration number:**

NCT02441426 (post-results) and NCT05121935.

**Strengths and limitations of this study**

- Well-established cohort with anthropometry measured at six time points over 11.5 years, permitting mixed-model regression analysis.
- Hormone assays and puberty assessments during early adolescence.
- Use of a validated measure of socioeconomic status that includes assessment of water access and toilet facilities.
- Limited by lack of data on nutritional intake.
- 6% of cohort lacked data on socioeconomic status.

## Introduction

 Childhood growth is a marker of overall health, with stunting—defined as a height-for-age z-score (HAZ) below −2 SD from the mean of the WHO Child Growth Standards—being a commonly used marker of inadequate growth. Stunting affects approximately 22% of children under 5 years globally,^1^ with consequences that can be profound and multifaceted, impacting physical health and being associated with cognitive development, educational attainment and economic productivity in adulthood.^2 3^

The aetiology of stunting is complex, involving potential contributions from inadequate nutrition, frequent infections, poor maternal health during pregnancy and lactation, and suboptimal caregiving practices—emphasising the importance of optimal linear growth over time.^4^ Enteric infections, in particular, play a critical role in growth faltering by impairing nutrient absorption and increasing metabolic demands.^5^,^8^ Recurrent infections and chronic inflammation can disrupt endocrine pathways crucial for growth, such as the insulin-like growth factor (IGF) axis, which is essential for normal growth and development.^9^ Prior studies have shown that children who experience stunting in early life are more likely to suffer from cognitive impairments, reduced school performance and lower economic productivity in adulthood,^3^ while children who experience catch-up growth—defined as accelerated growth following a period of growth faltering—may achieve taller stature, though the studies have been mixed on long-term effects on cognition.^10 11^

All of this raises the importance of understanding contributors to growth over time, growth deficits including stunting and underweight, and catch-up growth—knowledge that is needed for designing interventions to mitigate the long-term adverse effects of poor growth resulting in stunting.^12 13^ The Etiology, Risk Factors, and Interactions of Enteric Infections and Malnutrition and the Consequences for Child Health and Development (MAL-ED) study provides an excellent opportunity to evaluate these issues, as the study recruited a unique and comprehensive cohort from multiple sites in low- and middle-income countries to explore predictors of poor growth and development.^14^,^16^ The MAL-ED study followed children from birth through early childhood, collecting detailed data on growth, nutrition, infections and various biological markers.^16^ The site from Haydom in rural Tanzania has had follow-up assessments of cohort participants at ages 5, 8 and 11.5 years, allowing for a thorough investigation of the factors influencing growth trajectories and the potential for catch-up growth in stunted children. Haydom is a rural area in central Tanzania, located on a high plateau at approximately 1800 m above sea level.^14^ It is located in a low-resource area, with the majority of residents practising subsistence farming. The prevalence of stunting among members of the MAL-ED cohort was 70% at 18 months, compared with a prevalence of 43% in Tanzania overall at that time.^17^ While the Tanzanian Ministry of Health adheres to WHO policy recommendations for exclusive breastfeeding and minimum acceptable diet for young children, the proportion of children in MAL-ED who adhered to exclusive breastfeeding was only 55.9% by 30 days.^18^

In the current analysis, our goals were to identify key predictors of growth over time, catch-up growth and ongoing stunting among the Tanzanian cohort of MAL-ED from enrolment to 11.5 years—emphasising the factors that influence recovery from early-life deficits over time. We hypothesised that multiple factors—including socioeconomic status (SES; eg, maternal education, household income), hormones related to growth and metabolism (eg, IGF-1, growth hormone (GH), thyroid function) and the prevalence of early-life enteric pathogens—would influence a child’s growth over time and odds of stunting and catch-up growth. By understanding the determinants of growth, stunting and catch-up growth, we aim to inform interventions that can support recovery from early growth deficits and improve long-term health and development outcomes.

## Methods

### Study design and setting

This study focuses on the study site in Haydom, Tanzania, from the MAL-ED study, a longitudinal cohort investigation conducted across eight low- and middle-income countries.^14 15^ Funded by the Bill & Melinda Gates Foundation, the primary objective was to explore the intricate interactions between enteric infections, malnutrition, gut physiology, growth, cognitive development and immune responses in children from birth to 2 years old in resource-constrained settings. The trials were registered at ClinicalTrials.gov (NCT02441426 and NCT05121935). Written informed consent was obtained from the parents or guardians of all participants.

### Patient and public involvement

Patients or the public were not involved in the design, or conduct, or reporting, or dissemination plans of our research

### Participants

Newborns were enrolled within the first 14 days of life and followed through age 24 months, with follow-up studies performed at ages 5, 8 and 11.5 years. Inclusion criteria encompassed all live births within the study area, whereas exclusion criteria included birth weights below 1500 g, severe congenital anomalies, multiple births and maternal age under 16 years.

### Data collection

#### Anthropometric measurements

Anthropometric measurements were performed monthly from birth to 24 months, and subsequently at ages 5, 8 and 11.5 years. Weight was measured using digital scales calibrated weekly to ensure accuracy, and length and height were measured using standardised measurement boards and stadiometers. These measurements were performed by trained field workers following standardised protocols to minimise measurement errors. Two measurements were obtained; if these are within 1%, the average of the two was used; if these are not within 1%, a third measurement is obtained, and the average of the two closest measurements was used. Growth measurements were converted into weight-for-age z-score (WAZ), length-for-age z-score (LAZ; for children <2 years), HAZ (for children >2 years) and body mass index-for-age z-score (BMIZ) and weight-for-length (WFL) using the WHO growth curves.^19^ Stunting, low weight and low BMI were defined as LAZ/HAZ, WAZ and BMIZ <−2.0, respectively. In the absence of a universally accepted definition, catch-up growth was categorised in two different ways (analysed separately): (1) lack of stunting at 11.5 years (among those previously stunted at age 2 years) and (2) increase in HAZ by at least 0.5 from age 2 years to 11.5 years (ie, HAZ_11.5 years_–HAZ_2 years_).^20^

#### Environmental and socioeconomic factors

Environmental and socioeconomic data were collected at study entry and in follow-up at age 11.5 years using structured interviews with caregivers. Findings from the two time points were highly correlated, and values from 11.5 were used in regression models. Variables included maternal education, household income and a composite estimate of SES that had been derived across all MAL-ED sites based on improved Water and sanitation, Assets, Maternal education and household Income (WAMI) index.^21^ The water/sanitation portion included assessments of the family’s source of drinking water (eg, surface water, unprotected well, etc) and any available toilet facility.

#### Pubertal assessment

Because of the importance of pubertal development influencing linear growth in this peripubertal age range, we included in-person assessment of pubertal stage, acknowledging that this is intrusive for participants. Examinations were done by same-sex examiners whenever possible and with a parent present. Females were assessed for pubertal status based on breast contour using Tanner stages.^22^ Boys were assessed based on pubic hair status; however, during the study, it was found that any faint amount of body hair over the symphysis pubis had been inappropriately classified as pubic hair; therefore, for males, we could not distinguish between Tanner 1 and 2.

#### Stool testing

Stool samples were collected monthly and stored at −80 until time of testing. Procedures for sample extraction and testing have been previously detailed,^6 23 24^ and the protocols are available at dx.doi.org/10.17504/protocols.io.5qpvo3k8xv4o/v1. We used custom-designed TaqMan Array Cards (ThermoFisher, Carlsbad, California, USA) that compartmentalised probe-based quantitative PCR assays for 29 enteropathogens.^6 23 24^ Total pathogen burden was quantified as the average number of pathogens detected in non-diarrhoeal stools.

#### Hormone assays

Hormone levels were analysed at the University of Virginia Center for Research in the Reproduction Ligand Core Laboratory using the Siemens Immulite 2000. The lower limit of detection for assays was IGF-1 25 ng/mL; GH 0.05 ng/mL; free T4 0.3 ng/dL; thyroid-stimulating hormone (TSH) 0.002 μIU/mL; luteinizing hormone (LH) 0.1 μIU/mL; and follicle-stimulating hormone (FSH) 0.1 μIU/mL. For the purpose of mathematical analyses, individuals with measurements below the limit of detection were counted as having a value just below the lower limit of detection.

### Statistical analysis

All statistical analyses were conducted using SAS V.9.4. The growth-related outcomes of this study were: (1) anthropometry measures over time (LAZ/HAZ, WAZ and BMIZ measured at ages <2 weeks, and 2, 5, 8 and 11.5 years using mixed-model linear regression); (2) stunting, low weight and low BMI at ages 2, 5, 8 and 11.5 years using mixed-model logistic regression; and (3) catch-up growth, assessed separately as lack of stunting at 11.5 years (among those previously stunted at age 2 years) and as an increase in HAZ by at least 0.5 from age 2 years to 11.5 years (ie, HAZ_11.5 years_–HAZ_2 years_).^20^ For all mixed models, we incorporated random intercepts by participant, which accounts for the fact that repeated measurements or clustered observations within individual participants are not independent. All measures were assessed for normality and variables were log transformed where necessary. Hormone measures were standardised (with mean set to 0 and SD to 1.0) for regression analysis for improved direct comparison of the magnitude of association between hormones. Descriptive statistics, including mean, median, SD, minimum and maximum values, were calculated for key anthropometric measures, both overall and stratified by sex. Frequency distributions were assessed to determine the prevalence of stunting, low weight and low BMI among the cohort. Independent t-tests were performed to compare anthropometric measures between males and females. Relationships between dependent variables and explanatory variables were confirmed to be linear via visual inspection using scatterplots and assessment of residual plots. Longitudinal relationships between anthropometric measures at different ages and later predictors including SES factors and serum biomarkers were analysed through mixed-model linear regression models, which were adjusted for sex as a potential confounder. This included linear regression for anthropometry over time and logistic regression for odds of stunting, low weight, low BMI and catch-up growth. All models were run using all available data. Given the small size of this cohort, we considered statistical significance at p<0.05. However, because of the large number of potential predictors we assessed, as a sensitivity analysis, we applied a false discovery rate approach to determine which analyses would be considered statistically significant using this approach. For our analyses with 12 predictors, for example, p values were ranked in order, from smallest to largest, p(1)<p(2)<p(3)…<p(12). For each p value, statistical significance was determined by computing 0.05*i/14. Thus, significance for p(1) was set at 0.05*1/12=0.00416; p(2) was set at 0.05*2/12=0.00833, etc (online supplemental table 1). We then found the first p value where p(i) exceeded its calculated significance threshold value. All p values prior to this were declared ‘significant by false discovery rate approach’.^25^

## Results

### Participant characteristics

The study cohort comprised 182 children evenly distributed between males and females, with a mean age of 11.6 (SD=0.42) years at the most recent assessment. Table 1 details the demographic and SES characteristics of the participants. Because of significantly different early growth patterns between boys and girls in this area,^26^ these data are provided overall and by sex. In early life, males had a slightly higher burden of stool viral pathogens than females (0.36 vs 0.32, p=0.037). With the exception of slightly more schooling among females, there were no significant differences in SES between males and females (table 1). We lacked data to distinguish between Tanner 1 and 2 in boys, but when the stages Tanner 1 and 2 were combined for both males and females, there was no difference in prevalence of Tanner stage by sex.

**Table 1:** Participant demographics by sex*

Characteristic, mean (SD) unless otherwise noted	Overall (n=182)	Males	Females	P value for difference between males and females
Sex (n=182)	182	89 (48.9%)	93 (51.1%)	0.767
Age (years) (n=182)	11.60 (0.42)	11.59 (0.47)	11.61 (0.47)	0.661
Tanner stage (n=182)				
Tanner 1–2†	86 (males)	86		0.139†
Tanner 1	59 (females)		59 (63.4%)	
Tanner 2	26 (females)		26 (28.0%)	
Tanner 3	11	3	8 (8.6%)	
Menarche			0	
Child cumulative school attendance (years)(n=157)	3.68 (1.51)	3.39 (1.60)	3.93 (1.38)	**0.026**
Maternal education (years) (n=171)	5.10 (2.91)	5.18 (2.90)	5.02 (2.94)	0.724
Household income (US$/month) (n=171)	19 (11.4, 38)	19 (11.4, 38)	19 (11.4, 38)	0.997
WAMI (n=171)	0.236 (0.099)	0.203 (0.092)	0.218 (0.106)	0.350
Total stool pathogens‡ (n=168)	3.05 (0.50)	3.03 (0.43)	3.07 (0.56)	0.547
Total stool bacterial pathogens‡ (n=168)	2.16 (0.39)	2.14 (0.36)	2.19 (0.42)	0.411
Total stool viral pathogens‡ (n=168)	0.35 (0.13)	0.36 (0.13)	0.32 (0.13)	**0.037**

* Non-normally distributed variables are shown as median (IQR). Demographic variables that were skewed were compared using Wilcoxon rank-sum. P value <0.05 is indicated in bold.

† Due to systematic errors in data collection at the time of study, Tanner staging for males cannot be distinguished between Tanner 1 and 2. For χ2 test, females in Tanner 1 and 2 were also combined.

‡ Average per month.

WAMI, Water and sanitation, Assets, Maternal education and household Income.

### Growth measurements

**Figure 1:**
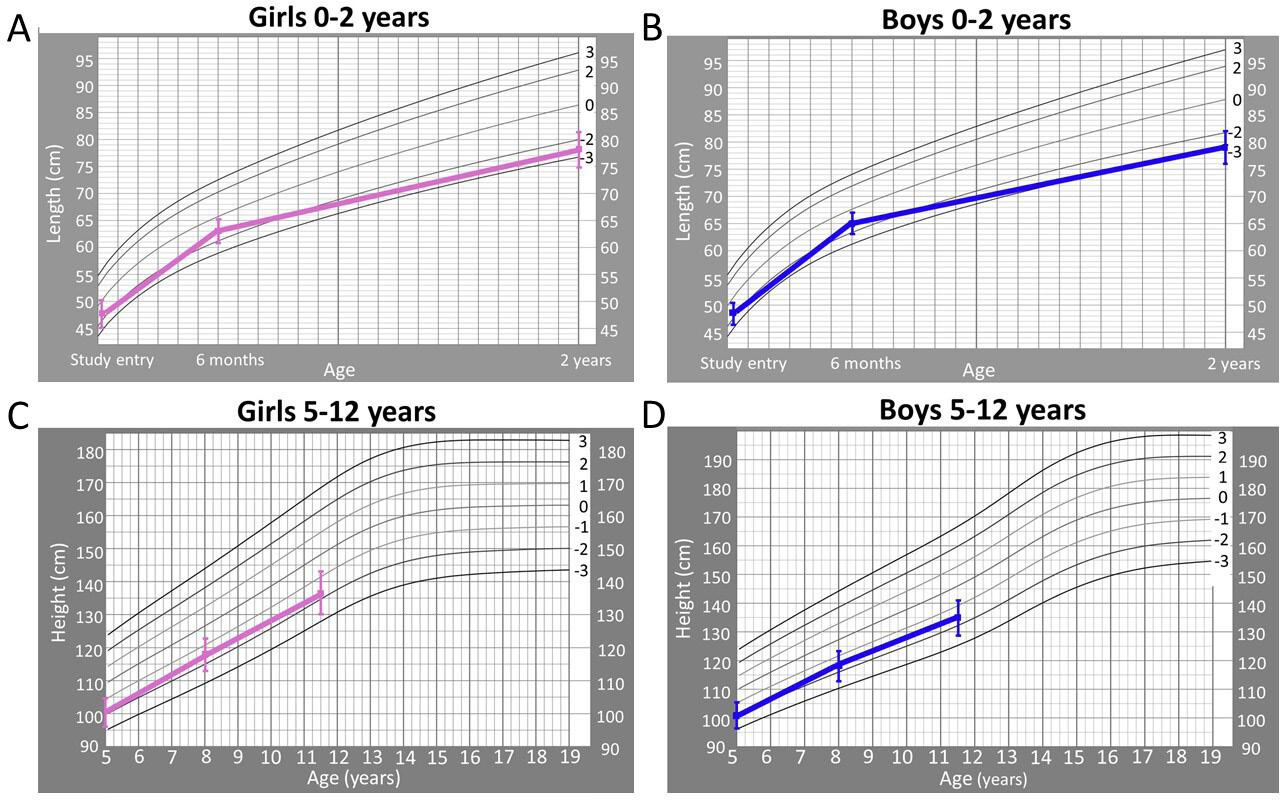
Mean length and height z-scores over time. Female (**A, C**) and male (**B, D**) length-for-age z-score and height-for-age z-score on WHO growth charts over time.

Table 2 provides longitudinal growth measurements overall and by sex, including z-scores and prevalence of those with z-score <−2 at enrolment (<2 weeks old), 6 months, 2 years, 5 years, 8 years and 11.5 years. Mean LAZ/HAZ for the cohort was significantly below the WHO mean from age 2 years through 11.5 years (figure 1).

**Table 2:** Growth measurements from study entry through age 11.5 years

	**Enrolment** (**≤2 weeks**) Mean (**SD**) **n=181**	**6 months** Mean (**SD**) **n=158**	**2 years** Mean (**SD**) **n=180**	**5 years** Mean (**SD**) **n=157**	**8 years** Mean (**SD**) **n=157**	Current (mean **11.5 years**) Mean (**SD**) **n=182**
**Overall**						
Length/height z-score	−1.05 (1.16)	−1.16 (0.924)	−2.70 (1.02)	−1.93 (0.939)	−1.52 (0.842)	−1.70 (0.914)
% stunting (LAZ/HAZ ≤−2)	16.4	16.4	72.6	47.9	29.5	39.0
Catch-up growth						
% stunted at 2 years old who were no longer stunted at age 11.5	N/A	N/A	N/A	N/A	N/A	48.5
% stunted at 2 years old who increased by 0.5 HAZ	N/A	N/A	N/A	59.3	78.5	78.5
Weight z-score	−0.164 (0.910)	−0.570 (1.13)	−1.31 (1.02)	−1.43 (0.882)	−1.45 (0.741)	−1.93 (0.768)
% below 3rd percentile	3.42	9.82	22.3	25.9	18.9	48.4
WFL/BMI z-score	0.751 (1.10)	0.419 (1.19)	0.122 (0.949)	−0.233 (0.943)	−0.697 (0.883)	−1.59 (0.720)
% wasted (≤−2)	1.15	1.84	0.61	3.70	6.75	24.7
**Males**						
Length/height z-score	−0.953 (1.15)	−1.23 (0.910)	−2.87 (1.05)*	−2.04 (0.932)	−1.65 (0.803)	−1.66 (0.867)
% stunting (≤−2)	11.2	19.1	75.0	49.4	31.2	38.2
Weight z-score	−0.144 (0.924)	−0.543 (1.19)	−1.37 (1.07)	−1.33 (0.832)	−1.44 (0.753)	−1.95 (0.776)
% below 3rd percentile	3.34	9.10	22.5	23.7	24.4	51.7
WFL/BMI z-score	0.643 (1.10)	0.573 (1.14)	0.184 (1.00)	0.019 (0.857)**	−0.536 (0.945)*	−1.48 (0.665)*
% wasted (≤−2)	1.14	0	0	1.3	6.49	19.1
**Females**						
Length/height z-score	−1.02 (1.17)	−1.12 (0.942)	−2.56 (0.965)*	−1.97 (0.955)	−1.41 (0.866)	−1.73 (0.961)
% stunting (≤−2)	19.6	17.2	71.7	47.5	27.5	39.8
Weight z-score	−0.137 (0.966)	−0.610 (1.10)	−1.28 (0.977)	−1.58 (0.910)	−1.47 (0.739)	−1.91 (0.765)
% below 3rd percentile	4.34	11.25	22.5	30.0	15.0	45.2
WFL/BMI z-score	0.770 (1.21)	0.268 (1.22)	0.03 (0.905)	−0.509 (0.963)**	−0.877 (0.803)*	−1.69 (0.757)*
% wasted (≤−2)	2.17	3.75	1.25	6.25	7.50	30.1

Parameters with a significant difference between males and females are indicated with an asterisk (*p<0.05, **p<0.001).

BMI, body mass index; HAZ, height-for-age z-score; LAZ, length-for-age z-score; WFL, weight-for-length z-score.

**Figure 2:**
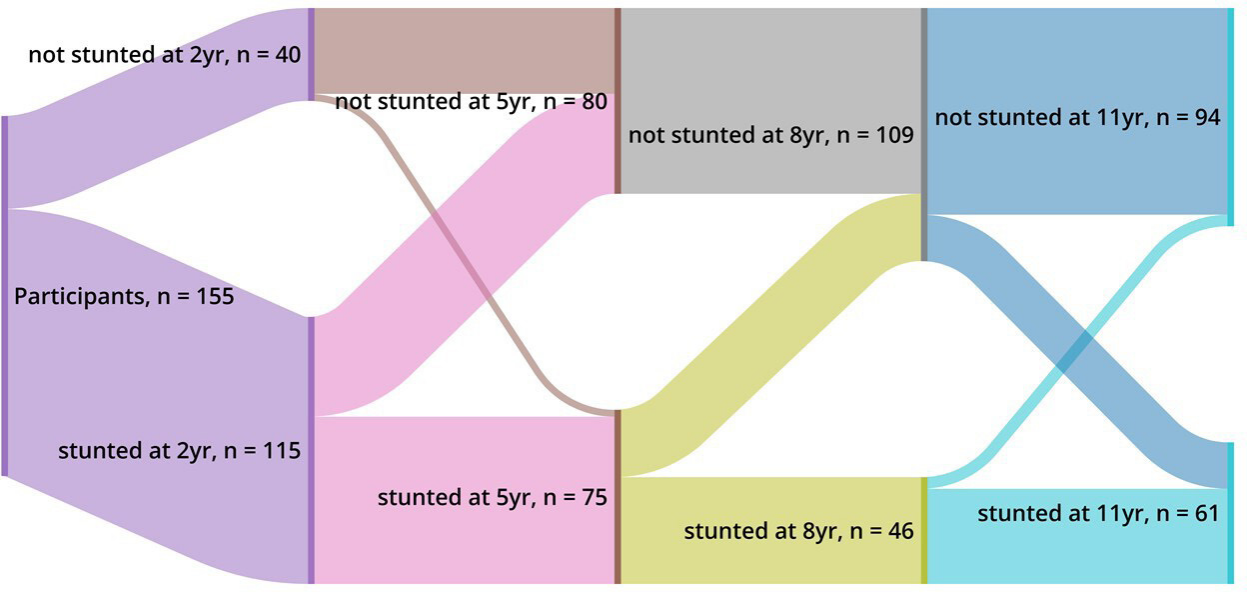
Sankey diagram illustrating proportion of participants with stunting by time point among the 155 participants who have growth measures at all five time points.

The prevalence of stunting was alarmingly high, peaking at 72.6% at 2 years of age. Although the percentage of stunted children decreased over time, it remained substantial, with 39.0% of participants classified as stunted at age 11.5 years (table 2). The Sankey diagram in figure 2 depicts the longitudinal trends in stunting from 2 years to 11.5 years among the 155 participants with growth measurements at all five time points (17 participants were missing measures at either 5 or 8 years)—illustrating the dynamic nature of stunting and catch-up growth within the cohort. Using a definition of catch-up growth as current resolution of stunting since age 2 years, 48.5% of those stunted in the cohort exhibited catch-up growth. Using a definition of catch-up growth of an increase in HAZ of at least 0.5 between 2 and 11.5 years, 78.5% of those stunted in the cohort exhibited catch-up growth (table 2).

### Hormone measurements

We assessed hormone levels at the 11.5-year time point as potential factors related to linear growth. We noted significant sex differences in several key biomarkers (table 3). Females had higher levels of IGF-1, GH and FSH compared with males. Specifically, IGF-1 levels were almost 40% higher in females compared with males (146.4±52.8 ng/mL vs 105.3±27.2 ng/mL, p<0.0001). GH levels were also higher in females compared with males (0.48 (0.18, 3.54) vs 0.24 (0.13, 0.56), p=0.001). FSH levels were significantly higher in females compared with males (2.15 (1.14, 3.34) vs 0.75 (0.48, 1.38), p<0.0001). TSH levels were not different by sex. If a TSH above 4.5 mIU/L is considered elevated,^27^ 54 participants (29.8%) were elevated, with the high value being 15 mIU/L.

**Table 3:** Hormone measurements at age 11.5 years*

n=182 for all measures	Overall	Males	Females	P value
IGF-1	15.6 (12.59, 19.09) nmol/L; 119.0 (96.3, 146) ng/mL Mean (SD): 16.5 (0.06) nmol/L; 126.3 (0.49) ng/mL	13.99 (11.14, 16.60) nmol/L; 107.0 (85.2, 127.0) ng/mL Mean (SD): 13.8 (3.56) nmol/L; 105.3 (27.2) ng/mL	18.04 (14.64, 21.70) nmol/L; 138.0 (112.0, 166.0) ng/mL Mean (SD): 19.1 (6.90) nmol/L; 146.4 (52.80) ng/mL	**<0.0001**
Growth hormone	0.34 (0.15, 0.71) μg/L; 0.34 (0.15, 0.71) ng/mL	0.24 (0.13, 0.56) μg/L; 0.24 (0.13, 0.56) ng/mL	0.48 (0.18, 3.54) μg/L; 0.48 (0.18, 3.54) ng/mL	**0.001**
Free T4	13.29 (12.38, 14.71) nmol/L; 1.03 (0.96, 14.71) μg/dL	13.16 (12.51, 14.71) nmol/L; 1.02 (0.97, 14.71) μg/dL	13.29 (12.26, 14.71) nmol/L; 1.03 (0.95, 14.71) μg/dL	0.88
TSH	3.29 (2.42, 4.76) mIU/L; 3.29 (2.42, 4.76) μIU/mL	3.30 (2.85, 4.76) mIU/L; 3.30 (2.85, 4.76) μIU/mL	3.26 (2.25, 4.65) mIU/L; 3.26 (2.25, 4.65) μIU/mL	0.179
LH	0.23 (0.12, 0.43) IU/L; 0.23 (0.12, 0.43) mIU/mL	0.21 (0.11, 0.39) IU/L; 0.21 (0.11, 0.39) mIU/mL	0.24 (0.12, 0.45) IU/L; 0.24 (0.12, 0.45) mIU/mL	0.646
FSH	1.24 (1.24, 2.67) IU/L; 1.24 (1.24, 2.67) mIU/mL	0.75 (0.48, 1.38) IU/L; 0.75 (0.48, 1.38) mIU/mL	2.15 (1.14, 3.34) IU/L; 2.15 (1.14, 3.34) mIU/mL	**<0.0001**
hsCRP	2.20 (2.20, 4.20) mg/L; 0.22 (0.22, 4.20) mg/dL	1.00 (1.00, 4.80) mg/L; 0.10 (0.10, 4.80) mg/dL	2.20 (1.00, 4.10) mg/L; 0.22 (0.10, 4.10) mg/dL	0.765

* Values are listed in both SI and standard units as median (IQR) with additional measure of mean (SD) given for normally distributed data. Skewed variables were compared using Wilcoxon rank-sum. P value <0.05 for difference between males and females is indicated in bold.

FSH, follicle-stimulating hormone; hsCRP, high-sensitivity C-reactive protein; IGF-1, insulin-like growth factor 1; LH, luteinizing hormone; SI, International System of Units; TSH, thyroid-stimulating hormone.

We next considered whether these differences in hormone levels may have been linked to pubertal status—potentially contributing to the observed sex differences in growth trajectories, with greater potential for recent catch-up growth in females. We lacked accurate pubertal assessments in males, but for females, the Tanner stage of breast development correlated with LH levels (regression estimate=0.12, SE=0.05, p=0.03). Further analysis revealed significant associations between IGF-1 levels and the tertiles of FSH and/or LH in both males and females. Specifically, in males, IGF-1 was positively associated with FSH tertiles (p=0.001) and LH tertiles (p=0.016). In females, similar associations were observed, with IGF-1 being positively associated with FSH tertiles (p=0.031) but not LH tertiles (p=0.101). This supports that higher IGF-1 levels in females may have been due to more advanced pubertal maturation.

### Predictors of growth, stunting and catch-up growth

The linear regression using a mixed-model analysis across multiple time points from 2 years to 11.5 years identified several significant factors associated with HAZ and WAZ over time (table 4). Maternal education, household income, WAMI and IGF-1 were positively associated with HAZ and WAZ when adjusting for participant sex. Specifically, IGF-1 showed a significant positive association with HAZ (parameter estimate 0.141±0.067, p=0.036) and WAZ (parameter estimate 0.127±0.056, p=0.024). Additionally, TSH was inversely associated with HAZ (parameter estimate −0.136±0.060, p=0.025) (table 4). We next assessed models that used all significant predictors together (with the exception of WAMI, which includes maternal education and family income in its calculation). In assessing predictors of LAZ/HAZ, maternal education, family income and IGF-1 remained significantly associated in the multivariable model, while TSH was no longer significantly associated (p=0.055) (table 4). For WAZ, each of the factors remained significant predictors.

**Table 4:** Mixed-model linear regression analysis for predictors of HAZ, WAZ and BMI*

Predictor	LAZ/HAZ		WAZ		BMIZ	
	Parameter estimate (SE)	P value	Parameter estimate (SE)	P value	Parameter estimate (SE)	P value
Maternal education	0.060 (0.021)	**0.003**	0.056 (0.017)	**0.001**	0.011 (0.017)	0.509
Family income (US$)	0.005 (0.001)	**<0.001**	0.005 (0.001)	**<0.001**	0.002 (0.001)	0.111
WAMI	0.268 (0.060)	**<0.0001**	0.237 (0.049)	**<0.0001**	0.066 (0.049)	0.176
IGF-1	0.141 (0.067)	**0.036** †	0.127 (0.056)	**0.024** †	0.027 (0.053)	0.606
GH	−0.087 (0.063)	0.165	−0.051 (0.052)	0.332	−0.006 (0.049)	0.900
Free T4	−0.042 (0.061)	0.493	−0.059 (0.051)	0.243	−0.022 (0.047)	0.649
TSH	−0.136 (0.060)	**0.025** †	−0.080 (0.051)	0.116	0.014 (0.047)	0.760
LH	−0.030 (0.062)	0.630	−0.043 (0.051)	0.401	−0.027 (0.048)	0.574
FSH	−0.088 (0.070)	0.210	−0.072 (0.058)	0.218	−0.018 (0.054)	0.744
Total stool pathogens	0.107 (0.062)	0.088	0.092 (0.052)	0.079	0.054 (0.050)	0.277
Total stool bacterial pathogens	0.064 (0.064)	0.312	0.074 (0.053)	0.160	0.062 (0.050)	0.219
Total stool viral pathogens	0.074 (0.063)	0.243	0.018 (0.053)	0.735	−0.057 (0.050)	0.252
Models with above significant predictors together‡						
Maternal education	0.062 (0.021)	**0.003**	0.053 (0.017)	**0.002**		
Family income (US$)	0.004 (0.001)	**0.002**	0.004 (0.001)	**<0.001**		
IGF-1	0.137 (0.065)	**0.037**	0.134 (0.053)	**0.013**		
TSH	−0.113 (0.059)	0.055				

* Parameter estimate and SE values were calculated using values normalised to the population mean for WAMI, GH, IGF-1, free T4, TSH, LH, FSH, total stool pathogens, total stool bacterial pathogens and total stool viral pathogens. Results are adjusted for participant’s sex. Log-transformed values were used for analysis of GH, free T4, TSH, LH and FSH. P value <0.05 is indicated in bold.

† Result is not considered statistically significant when false discovery rate approach is applied.

‡ WAMI was not included since maternal education and family income are major factors in calculating WAMI.

BMI, body mass index; BMIZ, BMI-for-age z-score; FSH, follicle-stimulating hormone; GH, growth hormone; HAZ, height-for-age z-score; IGF-1, insulin-like growth factor 1; LAZ, length-for-age z-score; TSH, thyroid-stimulating hormone; WAMI, Water and sanitation, Assets, Maternal education and household Income; WAZ, weight-for-age z-score.

The logistic regression analysis assessed for factors associated with stunting, low WAZ and low BMIZ (table 5), using a mixed-model approach from ages 2 years to 11.5 years. Significant predictors of stunting (HAZ/LAZ <−2) included years of maternal education (OR 0.986, 95% CI 0.813 to 0.950, p=0.001), household income (OR 0.986, 95% CI 0.979 to 0.994, p<0.001), WAMI (OR 0.003, 95% CI <0.001 to 0.031, p<0.0001), IGF-1 (OR 0.714, 95% CI 0.550 to 0.926, p=0.011) and TSH (OR 1.357, 95% CI 1.025 to 1.796, p=0.034). For low weight (WAZ <−2), maternal education, household income, WAMI and IGF-1 emerged as significant predictors. Interestingly, none of the predictors were significantly associated with low BMIZ, suggesting either that height and weight deficits were proportionate or that different factors may influence BMI compared with linear growth and weight. In assessing models that used all significant predictors together in the model (except WAMI which is calculated from maternal education and family income), for stunting, all of the predictors except TSH remained significant, while for low weight, each of the factors remained significant predictors.

**Table 5:** Mixed-model logistic regression analysis for predictors of stunting, low BMI and low weight*

Predictor	Stunting	P value	Low weight	P value	Low BMI	P value
	OR estimate (95% CI)		OR estimate (95% CI)		OR estimate (95% CI)	
Maternal education	0.879 (0.813 to 0.950)	**0.001**	0.888 (0.825 to 0.956)	**0.002**	0.955 (0.876 to 1.042)	0.298
Family income (US$)	0.986 (0.979 to 0.994)	**<0.001**	0.989 (0.982 to 0.997)	**0.005**	0.997 (0.990 to 1.004)	0.428
WAMI	0.563 (0.445 to 0.711)	**<0.0001**	0.606 (0.481 to 0.762)	**<0.0001**	0.791 (0.608 to 1.028)	0.080
IGF-1	0.714 (0.550 to 0.926)	**0.011**	0.712 (0.541 to 0.938)	**0.016**	0.753 (0.544 to 1.042)	0.087
GH	1.181 (0.933 to 1.493)	0.166	1.151 (0.916 to 1.446)	0.226	1.182 (0.913 to 1.531)	0.203
Free T4	1.084 (0.866 to 1.356)	0.483	0.984 (0.794 to 1.218)	0.879	1.079 (0.854 to 1.364)	0.521
TSH	1.357 (1.025 to 1.796)	**0.034** †	1.237 (0.945 to 1.618)	0.121	1.122 (0.837 to 1.504)	0.441
LH	1.054 (0.837 to 1.328)	0.653	0.985 (0.787 to 1.232)	0.892	0.965 (0.740 to 1.259)	0.793
FSH	1.155 (0.887 to 1.504)	0.286	1.004 (0.780 to 1.292)	0.977	0.992 (0.735 to 1.339)	0.958
Total stool pathogens	0.862 (0.676 to 1.100)	0.232	0.845 (0.662 to 1.079)	0.176	0.955 (0.728 to 1.252)	0.736
Total stool bacterial pathogens	0.885 (0.693 to 1.131)	0.328	0.878 (0.688 to 1.120)	0.294	0.983 (0.745 to 1.297)	0.905
Total stool viral pathogens	0.875 (0.681 to 1.124)	0.295	0.999 (0.778 to 1.284)	0.995	1.014 (0.766 to 1.341)	0.924
Models with above significant predictors together‡						
Maternal education	0.881 (0.817 to 0.951)	**0.001**	0.893 (0.830 to 0.960)	**0.002**		
Family income (US$)	0.988 (0.981 to 0.996)	**0.002**	0.991 (0.984 to 0.998)	**0.026**		
IGF-1	0.728 (0.567 to 0.935)	**0.013**	0.721 (0.554 to 0.938)	**0.015**		
TSH	1.423 (0.991 to 2.325)	0.058				

* Parameter estimate and SE values were calculated using values normalised to the population mean for WAMI, GH, IGF-1, free T4, TSH, LH, FSH (all measured at age 11.5 years), total stool pathogens (monthly through age 2 years), total stool bacterial pathogens and total stool viral pathogens. Results are adjusted for participant’s sex. Log-transformed values were used for analysis of GH, free T4, TSH, LH and FSH. P value <0.05 is indicated in bold.

† Result is not considered statistically significant when false discovery rate approach is applied.

‡ WAMI was not included since maternal education and family income are major factors in calculating WAMI.

BMI, body mass index; FSH, follicle-stimulating hormone; GH, growth hormone; IGF-1, insulin-like growth factor 1; LH, luteinizing hormone; TSH, thyroid-stimulating hormone; WAMI, Water and sanitation, Assets, Maternal education and household Income.

In assessing for catch-up growth, an analysis of children who were stunted at 2 years but not stunted at 11.5 years revealed IGF-1 as the only significant association (p=0.034) (table 6). When catch-up growth is defined at a change in HAZ of >0.5,^20^ 78.5% of participants exhibited catch-up growth from age 2 to 11.5, with maternal education, family income and WAMI remaining as significant predictors of this definition of catch-up growth (table 6).

**Table 6:** Odds of catch-up growth by age 11.5 years as defined by (1) lack of stunting at 1.51 years (among those previously stunted at age 2 years) or (2) increase in HAZ by at least 0.5 between ages 2 and 11.5 years*

Predictor	Stunted at age 2 years but not at age 11.5 years		HAZ increased by >0.2 between ages 2 and 11.5 years	
	OR (95% CI)	P value	OR (95% CI)	P value
Maternal education	1.068 (0.95 to 1.20)	0.275	0.87 (0.77 to 1.00)	**0.045** †
Family income (US$)	1.01 (0.995 to 1.025)	0.194	1.00 (0.99 to 1.00)	0.203
WAMI	15.8 (0.33 to 750)	0.161	0.02 (0.00 to 0.68)	**0.030** †
IGF-1	2.27 (1.32 to 3.88)	**0.002** †	2.02 (1.24 to 3.28)	**0.005** †
GH	1.37 (0.95 to 1.98)	0.096	1.22 (0.86 to 1.73)	0.268
Free T4	1.11 (0.80 to 1.54)	0.530	1.10 (0.79 to 1.55)	0.571
TSH	1.60 (0.96 to 2.66)	0.073	1.13 (0.83 to 1.56)	0.434
LH	0.94 (0.66 to 1.34)	0.734	1.46 (1.00 to 2.13)	0.050
FSH	0.90 (0.59 to 1.37)	0.623	1.45 (0.98 to 2.15)	0.063
Total stool pathogens	1.23 (0.58 to 2.59)	0.586	1.01 (0.99 to 1.03)	0.226
Stool bacterial pathogens	1.24 (0.49 to 3.13)	0.654	0.97 (0.48 to 1.97)	0.937
Stool viral pathogens	0.46 (0.02 to 13.44)	0.653	1.10 (0.44 to 2.73)	0.840
Models with above significant predictors together‡				
WAMI			0.01 (0.00 to 0.49)	**0.020**
IGF-1			2.23 (1.30 to 3.80)	**0.003**

* P value <0.05 is indicated in bold.

† Result is not considered statistically significant when false discovery rate approach is applied.

‡ When maternal education is used in the model instead of WAMI, estimate for maternal education is 0.88 (0.77, 1.00), p=0.056, and IGF-1 is 2.07 (1.24, 3.48), p=0.006.

FSH, follicle-stimulating hormone; GH, growth hormone; HAZ, height-for-age z-score; IGF-1, insulin-like growth factor 1; LH, luteinizing hormone; TSH, thyroid-stimulating hormone; WAMI, Water and sanitation, Assets, Maternal education and household Income.

Finally, to assess for whether higher IGF-1 levels were only associated with HAZ because of more advanced pubertal maturation, we assessed a model that included both IGF-1 and LH and FSH, revealing that following adjustment for gonadotropins, IGF-1 remained linked with HAZ/LAZ (parameter estimate 0.171±0.075, p=0.024) and WAZ (parameter estimate 0.157±0.063, p=0.012), as well as with odds of stunting as assessed for those stunted at 2 years but not 11.5 years (OR 2.37 (1.34, 4.17), p=0.003) and by HAZ increase 2–11.5 of >0.2 (OR 1.85 (1.13, 3.02), p=0.015).

While this study represented a small cohort, our assessment of multiple potential predictors presents the possibility of false positive values. As a sensitivity analysis, we applied a false discovery rate to assess whether these findings would be considered statistically significant when the multiple comparisons were taken into account. For several of the findings, the p values were above the level required to be considered statistically significant using the false discovery rate approach. We have indicated this in each of the tables.

## Discussion

This is the first study evaluating preadolescent follow-up of children from the MAL-ED birth cohort, exploring the predictors of growth over time, stunting and catch-up growth among children in the rural Tanzania site, with a particular focus on socioeconomic, hormonal and early-life infectious factors. This study is novel in including pubertal assessment and gonadotropin measures to account for the role of puberty in catch-up growth in this cohort. We noted a moderate amount of catch-up growth in that while 72.6% of children had stunting at age 2 years, only 29% were stunted at a mean age of 11.6 years—meaning that over 60% had experienced catch-up growth by that age. Our findings revealed that maternal education, household income, a composite estimate of SES and IGF-1 were positively associated with linear growth and negatively associated with odds of stunting, while only IGF-1 was associated with both definitions of catch-up growth. In considering the implications of these findings in the context of the literature, it is most likely that the estimates of SES represent upstream contributors to growth,^2 3^ while IGF-1 levels may be mixed in being both causal (eg, in individuals with a stronger genetic drive towards higher IGF-1)^28^ and correlational as an indirect marker of other contributors such as adequate nutrition and less inflammation.^29^ Overall, these findings highlight the multifactorial nature of growth in low-resource settings in early childhood.

The consistent associations between maternal education and household income with HAZ, stunting (inverse association) and catch-up growth align with existing research, underscoring the importance of SES as a causal agent for child growth and development.^2^ Maternal education likely enhances caregiving practices and health-seeking behaviours, contributing to improved growth outcomes.^3^ Furthermore, household income can impact the quality and quantity of nutrition and healthcare access, which are critical determinants of childhood growth.^13^ These findings suggest that interventions aimed at improving maternal education and household economic status could significantly impact child growth trajectories. Our composite SES variable WAMI also included variables related to water access, sanitation and hygiene (WASH), with WASH components being associated with childhood diarrhoea and stunting—though large-scale randomised trials to improve hygiene conditions failed to produce improvements in childhood growth.^30^

IGF-1, a critical growth factor, was significantly associated with all of our growth-related outcomes, which is not surprising, given its established role in promoting linear growth.^9^ IGF-1 mediates the effects of GH on bone growth and overall physical development, with additional associations with childhood cognitive development. However, in many cases, IGF-1 may be a surrogate for adequate nutrition and general health. Unfortunately, while inadequate nutrition is common in low- and middle-income countries^31^ and is clearly linked to poorer growth,^32^ it is also difficult to accurately assess nutrition intake in children, given cognitive limitations and reliance on parental observation and reporting.^33^ In particular, a nutrition questionnaire validated in this setting could have helped determine how much of the variance in IGF-1 levels relative to calorie intake per kilogram, for example. Without this, it is unclear how much the IGF-1 levels in this cohort were related to genetic versus nutritional factors.

It should also be noted that the observed associations between IGF-1 levels and increasing FSH/LH tertiles suggest that the elevated IGF-1 levels may also reflect earlier pubertal timing in some of these children at age 11.5 years. This relationship is potentially problematic for adult height potential in that early onset of puberty—marked by higher levels of hormones such as IGF-1, FSH and LH—leads to premature closure of epiphysial growth plates, ultimately leading to a reduction in adult height.^34 35^ Consequently, while higher IGF-1 levels might initially suggest a greater potential for better height growth by age 11.5 years, they could also reflect an advanced pubertal stage that restricts overall growth potential and reduces final adult height.^36^ To address this potential confounding issue, in our analysis we assessed regression models that adjusted for LH, FSH and IGF-1 levels, demonstrating that IGF-1 was still linked with HAZ/LAZ, supporting its relationship with height beyond its association with pubertal maturation. Nevertheless, future studies should incorporate assessments such as bone age or postpubertal anthropometry to further distinguish between these possibilities.

The significance of TSH being associated with HAZ suggests that thyroid function, often disrupted in malnourished children due to iodine deficiency, is an important factor in growth.^37^ Thyroid hormone regulates metabolism and energy balance, both essential for growth. These findings emphasise the need for assessing thyroid status in settings of growth failure—particularly in the first 3 years of life, when thyroid hormone is essential for normal brain development.^38^ Addressing these deficiencies could involve nutritional supplementation to improve the ultimate potential for catch-up growth in stunted children. Still, it should be noted that the majority of children in this cohort had a TSH in the normal range or only modestly elevated, and it is unclear how these minor variations may have been associated with growth outcomes. While height percentiles did not differ significantly between sexes, we noted higher levels of FSH, GH and IGF-1 in females—which may have been related to further progression through puberty among girls compared with boys. These all align with known biological patterns of earlier timing of puberty in females—which is usually accompanied by an earlier growth spurt as well. The higher levels of oestrogen over the course of puberty can also influence TSH regulation,^39^ though we did not find differences in TSH between sexes in this cohort.

While the total sum of enteric pathogens from early childhood did not emerge as a significant predictor in our study, the high prevalence of enteric infections in the cohort highlights the ongoing challenge of enteric disease in growth faltering.^8^ Previous studies have shown that recurrent infections can impair nutrient absorption and increase metabolic demands, contributing to stunting.^4^ It may be that these relationships are more pronounced early in life, as seen in the overall MAL-ED study,^6^ and that with age, other influences on growth predominate. Addressing the burden of enteric infections through improved sanitation, hygiene and access to clean water remains critical. These efforts could mitigate the impact of infectious diseases on growth, especially in resource-limited settings.

Our findings align with the broader literature on the determinants of childhood growth. The significant role of maternal education and household income in our study is consistent with global data linking socioeconomic factors to child health outcomes.^2^ The importance of IGF-1 and thyroid function has also been documented in other studies, reinforcing the biological pathways influencing growth.^9 37^ However, our study did not find significant associations with some factors, such as GH and free T4, which have been implicated in other research. This discrepancy could be due to differences in study design, population characteristics or the relatively small number of participants in this study. Future studies should explore these differences further to elucidate the complex interactions between various growth determinants. By identifying modifiable factors that contribute to catch-up growth, we can better target efforts to enhance child growth and development in high-burden settings, ultimately contributing to the broader goal of reducing the global burden of stunting and its long-term impacts.

The identification of significant socioeconomic and biological predictors of catch-up growth has important implications for public health interventions—which is particularly true to the extent that catch-up growth is related to improved developmental outcomes.^10^ Enhancing maternal education and improving household SES through targeted programmes can profoundly impact child growth outcomes. Policymakers should prioritise educational initiatives for mothers and economic support programmes for low-income families to address the root causes of stunting. Additionally, addressing nutritional deficiencies and hormonal imbalances through supplementation and healthcare interventions can support catch-up growth in stunted children. Interventions such as micronutrient supplementation, deworming and improved access to healthcare services are critical. These interventions can play a pivotal role in addressing the underlying causes of growth faltering.

While our study provides valuable insights, it has limitations that should be acknowledged. The observational nature of the study precludes causal inferences. Future research should explore the mechanistic pathways linking socioeconomic and biological factors to growth, potentially through longitudinal and interventional studies. We assessed multiple potential predictors, introducing the possibility for false discovery, though when a false discovery rate approach was applied, most of the findings remained statistically significant.^25^ Among our predictors, we had up to 7% missing data, limiting some analyses. We assessed breast development as a marker of pubertal status but did not have a reliable estimate of puberty (such as testicular size) in the boys, leaving us to rely on levels of gonadotropins, which are pulsatile in nature. Several of our predictors of catch-up growth were assessed using questionnaires/structured interviews, including important variables such as family income and maternal education. This introduces the potential for misclassification errors. We also lacked the means of assessing for differential misclassification, such as whether poorly grown children were more likely to provide inaccurate estimates, which would have made it appear that maternal education was associated with poorer growth, when in fact it was not. We also lacked estimates of nutrition intake; however, while adequate nutrition is clearly important for ongoing growth,^32^ it is also very difficult to accurately assess in childhood due to cognitive issues and reliance on proxy reporting^33^ and has not commonly been a part of assessments of catch-up growth.^10 11^ Moreover, the generalisability of our findings may be limited to similar socioeconomic and environmental contexts. Finally, the children reported on here have not reached adult height, so it is not clear whether the catch-up growth experienced by some will increase their ultimate adult height, which is important given the potential influence of puberty. Further research in diverse settings is also necessary to validate and extend our findings. Investigating the role of genetic factors and environmental exposures could provide a more comprehensive understanding of growth determinants. Understanding these interactions could lead to more targeted and effective interventions.

In conclusion, this study underscores the critical role of both socioeconomic and biological factors in predicting ongoing growth and catch-up growth in children. By identifying modifiable predictors, our findings contribute to developing targeted interventions aimed at improving growth outcomes in resource-limited settings. Continued research and comprehensive interventions are essential to reduce the global burden of stunting and promote healthy growth and development in children. Our findings provide insights into the complex dynamics of child growth and offer a foundation for future research and policy development.

## Supplementary material

10.1136/bmjopen-2025-100955 online supplemental table 1

## Data Availability

Data are available upon reasonable request.
