# Supplementary material for: Catch-up growth following early-life stunting in a low-resource area in rural Tanzania: the MAL-ED Metabolic study
Source: BMJ Open. 2025 Aug 21;15(8):e100955. doi: 10.1136/bmjopen-2025-100955 (PMC12374642; doi:10.1136/bmjopen-2025-100955)
Supplement: online supplemental table 1 [file bmjopen-15-8-s001.docx]

**MAL-ED Metabolic Predictors of Stunting: Supplemental Material**

Supplementary Table 1: P-value cut-offs for false discovery rate approach.*

| For analyses involving 3 different outcomes | | For analyses involving 4 different outcomes | | For analyses involving 12 different outcomes | |
| --- | --- | --- | --- | --- | --- |
| Sequential P value order | False discovery rate P value cut off | Sequential P value order | False discovery rate P value cut off | Sequential P value order | False discovery rate P value cut off |
| 1 | 0.01667 | 1 | 0.0125 | 1 | 0.004167 |
| 2 | 0.03333 | 2 | 0.025 | 2 | 0.008333 |
| 3 | 0.05 | 3 | 0.0375 | 3 | 0.0125 |
|  |  | 4 | 0.05 | 4 | 0.016667 |
|  |  |  |  | 5 | 0.020833 |
|  |  |  |  | 6 | 0.025 |
|  |  |  |  | 7 | 0.029167 |
|  |  |  |  | 8 | 0.033333 |
|  |  |  |  | 9 | 0.0375 |
|  |  |  |  | 10 | 0.041667 |
|  |  |  |  | 11 | 0.045833 |
|  |  |  |  | 12 | 0.05 |

* As a sensitivity analysis, for each analysis (for each set of outcomes that included multiple potential predictors), P values were ranked from lowest to highest and compared to the P value cut-off’s shown in order here. An analysis result was only considered statistically significant if it was lower than the corresponding false discovery rate P value cut-off (and if all previous P values from the sequential order for that analysis were also lower than their corresponding false discovery rate P value).
