# Supplementary material for: Catch-up growth following early-life stunting in a low-resource area in rural Tanzania: the MAL-ED Metabolic study
Source: BMJ Open. 2025 Aug 21;15(8):e100955. doi: 10.1136/bmjopen-2025-100955 (PMC12374642; doi:10.1136/bmjopen-2025-100955)
Supplement: Supplementary file 2 [file bmjopen-2025-100955.reviewer_comments.pdf]

PEER REVIEW HISTORY

BMJ Open publishes all reviews undertaken for accepted manuscripts. Reviewers are asked to complete a checklist review form and are provided with free text boxes to elaborate on their assessment. These free text comments are reproduced below.

ARTICLE DETAILS

Title (Provisional)

Catch-up Growth Following Early Life Stunting in a Low-Resource Area in Rural Tanzania: The MAL-ED Metabolic Study

Authors

Nemati, Kiya; Michael, Yotham Z.; Hhando, Bernadetha P.; Jatosh, Samwel; Houpt, Eric R.; Mduma, Estomih; DeBoer, Mark Daniel

---

VERSION 1 - REVIEW

**Reviewer**	**1**
**Name**	**Luo, Xiaoping**
**Affiliation**	**Huazhong Univ Sci**
**Date**	**31-Mar-2025**
**COI**	**None**

The study aimed to identify key predictors of growth over time, stunting, and catch-up growth among children in rural Tanzania. It was a relative large cohort with a long follow-up time. The analyses were well-conducted. However, when discuss the significance of TSH, the authors should be more careful. Although the detail resutls were not shown, it looked like the TSH levels were still in the normal range. In this case, a slightly difference in TSH level does not refelct a change in thyroid function, which means it is not clinical significant.

**Reviewer**	**2**
**Name**	**Ataullahjan, Anushka**
**Affiliation**	**Western University**
**Date**	**01-Apr-2025**
**COI**	**None**

Thank you for the opportunity to review this very interesting and important work. I have a few minor comments that I believe would enrich this paper

1- Contextualizing the study in Tanzania would be useful to readers, please give an introduction to the context with a short overview of stunting rates, and policies in place (this may also help readers understand why in some cases certain findings do not align with dominant literature)

2-Note gender differences is used when I believe sex differences is intended

3- The discussion is very detailed and interesting, but much of what is presented is how the study aligns with previous research, what is new beyond the methodological approach? What does this study add?

4-There seems to be no description of WASH in the findings although mentioned in abstract, please clarify further

---

Reviewer 3

Name Saez, Marc

Affiliation Universitat de Girona, Research Group on Statistics, Econometrics and Health (GRECS)

Date 10-May-2025

COI None

The authors attempted to identify key predictors of growth over time, stunting, and catch-up growth in children in rural Tanzania. However, the authors were only partially successful in achieving their goal. In fact, I have a few comments, most of them major.

Major comments

1.- The authors indicate they use mixed models, but where are the random effects? In the independent term?

The authors should explain this in detail.

2.- Why isn't logistic regression mixed? Or is it? The authors should explain all this in detail.

3.- The relationships between the dependent variable and the explanatory variables do not have to be linear. For example, anthropometry, but not only that. In fact, it is very likely that they are not.

Authors should provide evidence that the relationship is linear or nonlinear, and in the latter case, model that nonlinear relationship.

4.- A very important limitation is the lack of estimates of nutritional intake.

The authors should explain this in much more detail. They should point out the effects on their results, how they could have avoided them, and if not, why.

5. - Among the limitations, the authors should point out that important predictors, such as environmental and socioeconomic factors, were collected using structured interviews. This means that measurement errors (i.e., misclassification) could occur.

The authors should discuss this limitation in detail, indicate whether it is differential or non-differential, including using statistics (e.g., whether these variables are correlated with any other variables, etc.), and indicate how this error would influence their results.

Minor comments

1.- In addition to the median, authors should use the interquartile range (or Q1 and Q3) when summarizing variables.

Reviewer **4**

Name **Shamsutdinova, Diana**

Affiliation **King's College London, Biostatistics and Health Informatics**

Date **20-May-2025**

COI **None**

Review of “Catch-up Growth Following Early Life Stunting in a Low-Resource Area in Rural Tanzania: The MAL-ED Metabolic Study” for BMJ Open.

This manuscript addresses an important question, investigating the influence of socio-economic factors (such as maternal education, WAMI index, and household income) on early childhood physical growth and catch-up growth in children who exhibited stunting at age 2 but not at 11.5 years in rural and low-resource areas. The study's focus on both environmental and biological factors is commendable and contributes to the literature on early childhood development and health disparities. I also appreciate the descriptive tables and Figure2 clearly showing the developmental paths in the cohort. However, several methodological and interpretative issues should be addressed before potential publication.

My key concern is the treatment of biological markers (e.g., IGF-1, GH, TSH) and socio-economic indicators as if they are of equal causal weight. While socio-economic factors such as maternal education and household income may imply causal influence on physical growth, the associations with hormonal levels are likely to be correlational. For instance, one would expect to see higher IGF-1 levels in those with better growth outcomes, though this does not tell much about why that happened. The manuscript would benefit from clearer articulation of these distinctions. Specifically, interpretations should clarify, for example, whether the association with maternal education remains significant after adjustment for hormonal levels, and what such findings imply about causal pathways. Similarly, the results in Table 6, where socio-economic factors are not significant factors for catch-up growth while IGF-1 is, could suggest that measured socio-economic factors did not contribute to the

increased IGF-1, which indicates the influence of other unmeasured variables, for example, genetic predisposition, overall health status, or psychosocial factors. Alternatively, if hormonal markers are being used as proxies (in part) for nutritional status or other mediating variables, this assumption should be explicitly stated and discussed.

Secondly, the manuscript conducts a large number of statistical tests on a relatively modest sample size (n = 180). Without adjustment for multiple comparisons, the risk of false positives is very high. It is strongly recommended that the authors apply an appropriate correction for multiple testing (e.g., Bonferroni), and re-evaluate the statistical significance of the results. Notably, associations involving IGF-1 and TSH may no longer reach significance after corrections, and this should be transparently reported and interpreted.

Given the substantial differences observed in certain hormone levels between boys and girls, a more in-depth discussion seems to be needed. For example, TSH levels are typically higher in females, and estrogen can influence TSH regulation. Similarly, FSH levels differ by sex and developmental stage, and also was noticed to be higher in women. The authors should discuss whether the observed differences align with known biological patterns.

The manuscript does not provide information regarding the extent of missing data or the methods used to address it, please add relevant information.

Finally, the assessment of pubertal status in children, particularly if it involved physical examination, may be considered intrusive relative to the primary aims of the study. Perhaps, the authors should clarify the necessity and ethical justification of this component.

VERSION 1 - AUTHOR RESPONSE

Reviewer: 1
Dr. Xiaoping Luo, Huazhong Univ Sci
Comments to the Author:
The study aimed to identify key predictors of growth over time, stunting, and catch-up growth among children in rural Tanzania. It was a relatively large cohort with a long follow-up time. The analyses were well-conducted. However, when discuss the significance of TSH, the authors should be more careful. Although the detail results were not shown, it looked like the TSH levels were still in the normal range. In this case, a slightly difference in TSH level does not reflect a change in thyroid function, which means it is not clinical significant.

RESPONSE: Thank you—we agree that these variations in TSH value were minor and of unclear significance. We have addressed this by:

a. Clarifying the distribution of TSH elevations: "If a TSH above 4.5 mIU/L is considered elevated, 54 participants (29.8%) were elevated, with the high value being 15 mIU/L." (Results, page 14, paragraph 1, line 7).
b. Softening our language regarding the associations between TSH and growth outcomes (Discussion, page 23, paragraph 3, line 1).

c. Adding this sentence: "Still, it should be noted that the majority of children in this cohort had a TSH in the normal range or only modestly elevated, and it is unclear how these minor variations may have been associated with growth outcomes." (Discussion, page 23, paragraph 3, line 6).

Reviewer: 2
Dr. Anushka Ataullahjan, Western University
Comments to the Author:
Thank you for the opportunity to review this very interesting and important work. I have a few minor comments that I believe would enrich this paper

1- Contextualizing the study in Tanzania would be useful to readers, please give an introduction to the context with a short overview of stunting rates, and policies in place (this may also help readers understand why in some cases certain findings do not align with dominant literature)

RESPONSE: We have added an introduction to these issues, including how the Haydom area compares to Tanzanian national rates of stunting in this age range, and how the MAL-ED cohort overall had poor adherence to national policies to encourage exclusive breastfeeding: "Haydom is a rural area in central Tanzania, located on a high plateau at approximately 1,800 meters above sea level. It is located in a low-resource area, with the majority of residents practicing subsistence farming. The prevalence of stunting among members of the MAL-ED cohort was 70% at 18 months, compared to a prevalence of 43% in Tanzania overall at that time. While the Tanzanian Ministry of Health adheres to WHO policy recommendations for exclusive breast feeding and minimum acceptable diet for young children, the proportion of children in MAL-ED who adhered to exclusive breastfeeding was only 55.9% by 30 days." (Introduction, page 6, paragraph 1, line 4.)

2-Note gender differences is used when I believe sex differences is intended

RESPONSE: Thank you—we have corrected these instances.

3- The discussion is very detailed and interesting, but much of what is presented is how the study aligns with previous research, what is new beyond the methodological approach? What does this study add?

RESPONSE: We have better highlighted the novelty in this report, including the extent of catch-up growth (over 60% of those previously stunted) and the assessment of both pubertal stage and gonadotropins to account for the role of puberty in this peri-pubertal cohort: "This study is novel in including pubertal assessment and gonadotropin measures to account for the role of puberty in catch-up growth in this cohort. We noted a moderate amount of catch-up growth in that while 72.6% of children had stunting at age 2 years, only 29% were stunted at a mean age of 11.6 years—meaning that over 60% had experienced catch up growth by that age." (Discussion, page 21, paragraph 4, line 3.)

4-There seems to be no description of WASH in the findings although mentioned in abstract, please clarify further

RESPONSE: Thank you—we have added description of the WASH assessments that are included in the composite WAMI variable: “The water/sanitation portion included assessments of the family’s source of drinking water (e.g. surface water, unprotected well, etc.) and any available toilet facility.” (Methods, page 8, paragraph 2, line 6.)

Also, in the Discussion we have added the following regarding associations of WASH with growth outcomes: “Our composite SES variable WAMI also included variables related to water access, sanitation and hygiene (WASH), with worsened sanitation components being associated with childhood diarrhea and stunting—though largescale randomized trials to improve hygiene conditions failed to produce improvements in childhood growth” (Discussion, page 22, paragraph 2, line 7).

Reviewer: 3
Dr. Marc Saez, Universitat de Girona, CIBER of Epidemiology and Public Health (CIBERESP)
Comments to the Author:

The authors attempted to identify key predictors of growth over time, stunting, and catch-up growth in children in rural Tanzania. However, the authors were only partially successful in achieving their goal. In fact, I have a few comments, most of them major.
Major comments

1.- The authors indicate they use mixed models, but where are the random effects? In the independent term?
The authors should explain this in detail.

RESPONSE: Yes, sorry--both the mixed model linear and logistic regressions include random intercepts by participant. We have added this to the Methods:

“For all mixed models, we incorporated random intercepts by participant, which accounts for the fact that repeated measurements or clustered observations within individual participants are not independent.” (Methods, page 9, paragraph 3, line 6.)

2.- Why isn't logistic regression mixed? Or is it? The authors should explain all this in detail.

RESPONSE: The logistic regression models assessing for stunting, low weight and low BMI were indeed mixed models. This is now clarified in the Methods: “stunting, low weight and low BMI at age 2, 5, 8 and 11.5 years using mixed-model logistic regression” (Methods, page 9, paragraph 3, line 4.)

3.- The relationships between the dependent variable and the explanatory variables do not have to be linear. For example, anthropometry, but not only that. In fact, it is very likely that they are not. Authors should provide evidence that the relationship is linear or nonlinear, and in the latter case, model that nonlinear relationship.

RESPONSE: Thank you--we have performed linear regression plots and assessed the scatterplot and plots of residuals between all of our outcomes and explanatory variables to assess for any non-linear relationships. This is now stated in the Methods: “Relationships between dependent variables and explanatory variables were confirmed to be linear via visual inspection using scatterplots and assessment of residual plots.” (Methods, page 24, paragraph 1, line 4.)

4.- A very important limitation is the lack of estimates of nutritional intake.
The authors should explain this in much more detail. They should point out the effects on their results, how they could have avoided them, and if not, why.

RESPONSE: We agree with this significant limitation—though the accurate assessment of nutrition intake in children is difficult—and may be more difficult in this setting, where exact volumes and weights are not commonly used in households to determine quantities of food intake. We now acknowledge this limitation as follows: "Unfortunately, while inadequate nutrition is common in low- and middle-income countries and is clearly linked to poorer growth, it is also difficult to accurately nutrition intake in children, given cognitive limitations and reliance on parental observation and reporting. In particular, a nutrition questionnaire validated in this setting could have helped determine how much of the variance in IGF-1 levels relative to calorie intake per kilogram, for example. Without this, it is unclear how much the IGF-1 levels in this cohort were related to genetic vs. nutritional factors." (Discussion, page 22, paragraph 3, line 5).

5. - Among the limitations, the authors should point out that important predictors, such as environmental and socioeconomic factors, were collected using structured interviews. This means that measurement errors (i.e., misclassification) could occur.
The authors should discuss this limitation in detail, indicate whether it is differential or non-differential, including using statistics (e.g., whether these variables are correlated with any other variables, etc.), and indicate how this error would influence their results.

RESPONSE: Thank you—we now acknowledge this in the Limitations section: "Several of our predictors of catch-up growth were assessed using questionnaires/structured interviews, including important variables such as family income and maternal education. This introduces the potential for misclassification errors. We also lacked the means of assessing for differential misclassification, such as whether poorly-grown children were more likely to provide inaccurate estimates, which would have made it appear that maternal education was associated with poorer growth, when in fact it was not." (Discussion, page 25, paragraph 2, line 8.)

Minor comments

1.- In addition to the median, authors should use the interquartile range (or Q1 and Q3) when summarizing variables.

RESPONSE: Thank you--we now provide median (IQR) for all of the hormones (and additionally provide mean (SD) for those that are normally distributed. We also apologize for the inconsistency in how intraquartile range was expressed for non-normally-distributed variables, in that in some places we provided this with a comma separating the Q1 and Q3 values, while in others a dash was used between them. We have now made consistent use of commas separating them. For normally-distributed variables, we continue to provide mean and standard deviation.

Reviewer: 4
Dr. Diana Shamsutdinova, King's College London
Comments to the Author:
Review of "Catch-up Growth Following Early Life Stunting in a Low-Resource Area in Rural Tanzania: The MAL-ED Metabolic Study" for BMJ Open.

This manuscript addresses an important question, investigating the influence of socio-economic factors (such as maternal education, WAMI index, and household income) on early childhood physical growth and catch-up growth in children who exhibited stunting at age 2 but not at 11.5 years in rural and low-resource areas. The study's focus on both environmental and biological factors is commendable and contributes to the literature on early childhood development and health disparities. I also appreciate the descriptive tables and Figure2 clearly showing the developmental paths in the cohort. However, several methodological and interpretative issues should be addressed before potential publication.

1a. My key concern is the treatment of biological markers (e.g., IGF-1, GH, TSH) and socio-economic indicators as if they are of equal causal weight. While socio-economic factors such as maternal education and household income may imply causal influence on physical growth, the associations with hormonal levels are likely to be correlational. For instance, one would expect to see higher IGF-1 levels in those with better growth outcomes, though this does not tell much about why that happened. The manuscript would benefit from clearer articulation of these distinctions. Specifically, interpretations should clarify, for example, whether the association with maternal education remains significant after adjustment for hormonal levels, and what such findings imply about causal pathways. Similarly, the results in Table 6, where socio-economic factors are not significant factors for catch-up growth while IGF-1 is, could suggest that measured socio-economic factors did not contribute to the increased IGF-1, which indicates the influence of other unmeasured variables, for example, genetic predisposition, overall health status, or psychosocial factors. Alternatively, if hormonal markers are being used as proxies (in part) for nutritional status or other mediating variables, this assumption should be explicitly stated and discussed.

RESPONSE: Thank you for this excellent point. We have performed analyses that incorporate both the significant socio-economic and hormone factors for linear regression with LAZ/HAZ and WAZ and logistic regression for stunting and low weight and catch-up growth, reporting persistent associations for these factors the in combined models (except for TSH and the slight exception in the model for LAZ/HAZ, for which IGF-1 was reduced to p=0.05)(see Tables 4-6). We further expand our Discussion of these points:

"In considering the implications of these findings in the context of the literature, it is most likely that the estimates of SES represent upstream contributors to growth, while IGF-1 levels may be mixed in being both causal (e.g. in individuals with a stronger genetic drive toward growth hormone release) and correlational as an indirect marker of other contributors such as adequate nutrition and less inflammation." (Discussion, page 22, paragraph 1, line 1.)

"The consistent associations between maternal education and household income with HAZ, stunting (inverse association) and catch-up growth align with existing research, underscoring the importance of SES as a causal agent for child growth and development." (Discussion, page 22, paragraph 2, line 2.)

"However, in many cases IGF-1 may be a surrogate for adequate nutrition and general health." (Discussion, page 22, paragraph 3, line 4).

2. Secondly, the manuscript conducts a large number of statistical tests on a relatively modest sample size (n = 180). Without adjustment for multiple comparisons, the risk of false positives is very high. It is strongly recommended that the authors apply an appropriate correction for multiple testing (e.g., Bonferroni), and re-evaluate the statistical significance of the results. Notably,

associations involving IGF-1 and TSH may no longer reach significance after corrections, and this should be transparently reported and interpreted.

RESPONSE: As a sensitivity analysis, we have applied a false discovery approach to the interpretation of the data, which is reflected for each of the tables involving assessment of multiple potential predictors. This is now reflected in the Methods:

"Because of the large number of potential predictors we assessed, as a sensitivity analysis we applied a false discovery rate approach to determine which analyses would be considered statistically significant using this approach. For our analyses with 12 predictors (for example), p values were ranked in order, smallest to largest, p[1] < p[2] < p[3]…. < p[12]. For each p value, statistical significance was determined by computing 0.05*i/14. Thus, significance for p[1] was set at 0.05*1/12 = 0.00416; p[2] was set at 0.05*2/12 = 0.00833, etc. (Supplementary Table 1f). We then found the first p value where p[i] exceeded its calculated significance threshold value. All P values prior to this were declared "significant by false discovery rate approach." (Methods, page 10, paragraph 1, line 10.)

And the Results:

"While this study represented a small cohort, our assessment of multiple potential predictors presents the possibility of false positive values. As a sensitivity analysis, we applied a false discovery rate to assess whether these findings would be considered statistically significant when the multiple comparisons were taken into account. For several of the findings, the p values were above the level required to be considered statistically significant using the false discovery rate approach. We have indicated this in each of the tables." (Results, page 21, paragraph 3, line 1).

And the Limitations:

"We assessed multiple potential predictors, introducing the possibility for false discovery, though when a false discovery rate approach was applied, most of the findings remained statistically significant." (Discussion, page 25, paragraph 2, line 4.)

3. Given the substantial differences observed in certain hormone levels between boys and girls, a more in-depth discussion seems to be needed. For example, TSH levels are typically higher in females, and estrogen can influence TSH regulation. Similarly, FSH levels differ by sex and developmental stage, and also was noticed to be higher in women. The authors should discuss whether the observed differences align with known biological patterns.

RESPONSE: Thank you—we have added the following paragraph: "While height percentiles did not differ significantly between sexes, we noted higher levels of FSH, growth hormone and IGF-1 in females—which may have been related to further progression through puberty among girls compared to boys. These all align with known biological patterns of earlier timing of puberty in females—which is usually accompanied by an earlier growth spurt as well. The higher levels of estrogen over the course of puberty can also influence TSH regulation, though we did not find differences in TSH between sexes in this cohort." (Discussion, page , paragraph , line).

4. The manuscript does not provide information regarding the extent of missing data or the methods used to address it, please add relevant information.

RESPONSE: In Tables 1-3 we now list the number of participants for whom there are data available for each of the variables used. We have added to the Methods that, “All models were run using all available date” (Methods, page , paragraph , line).

We also acknowledge the missing data in the Limitations: “Among our predictors we had up to 7% missing data, limiting some analyses” (Discussion, page 23, paragraph 4, line 1).

5. Finally, the assessment of pubertal status in children, particularly if it involved physical examination, may be considered intrusive relative to the primary aims of the study. Perhaps, the authors should clarify the necessity and ethical justification of this component.

RESPONSE: Thank you. We have acknowledged this as follows: “Because of the importance of pubertal development influencing linear growth in this peri-pubertal age range, we included in-person assessment of pubertal stage, acknowledging that this is intrusive for participants. Exams were done by same-sex examiners whenever possible and with a parent present.” (Methods, page 8, paragraph 3, line 1.)

VERSION 2 - REVIEW

Reviewer **3**

Name **Saez, Marc**

Affiliation **Universitat de Girona, Research Group on Statistics, Econometrics and Health (GRECS)**

Date **20-Jul-2025**

COI

The authors have responded very well not only to my comments but also to those of the other reviewers. They have also included many of them in the new version of the manuscript. I have no further comments.

Reviewer **4**

Name **Shamsutdinova, Diana**

Affiliation **King's College London, Biostatistics and Health Informatics**

Date **28-Jul-2025**

COI

Thank you for addressing the issues raised in the first review. In particular, I appreciate the clarification of the hormonal association being explained as either the genetic predisposition to high height or a proxy for the quality of nutrition and other socio-economic factors.
